# Correlation of Circulating Glucocorticoid-Induced TNFR-Related Protein Ligand Levels with Disease Activity in Patients with Systemic Lupus Erythematosus

**DOI:** 10.1155/2012/265868

**Published:** 2012-12-04

**Authors:** Lei Gu, Lingxiao Xu, Xiaojun Zhang, Wenfeng Tan, Hong Wang, Miaojia Zhang

**Affiliations:** ^1^Department of Rheumatology, The First Affiliated Hospital of Nanjing Medical University, 300 Guangzhou Road, Nanjing 210029, China; ^2^Department of Respiratory Disease, The First Affiliated Hospital of Nanjing Medical University, 300 Guangzhou Road, Nanjing 210029, China

## Abstract

The aim of this paper is to investigate the correlation of glucocorticoid-induced tumor necrosis factor receptor- (TNFR-) related protein ligand (GITRL) with disease activity and organ involvement in patients with systemic lupus erythematosus (SLE). Serum GITRL levels were measured in 58 patients with SLE and 30 healthy controls matched for age and sex. Patients were assessed for clinical and laboratory variables. Correlations of serum GITRL levels with SLEDAI, laboratory values, and clinical manifestations were assessed. Serum GITRL levels were determined by ELISA. Serum GITRL levels were markedly increased in patients with SLE compared with healthy controls (mean 401.3 ng/mL and 36.59 ng/mL, resp.; *P* < 0.0001). SLE patients with active disease showed higher serum GITRL levels compared to those with inactive disease (mean 403.3 ng/mL and 136.3 ng/mL, resp; *P* = 0.0043) as well as normal controls (36.59 ng/mL; *P* < 0.0001). Serum GITRL levels were positively correlated with SLEDAI, titers of anti-dsDNA antibody, erythrocyte sedimentation rate (ESR), and IgM and negatively correlated with complement3 (C3). Serum GITRL levels were higher in SLE patients with renal involvement and vasculitis compared with patients without the above-mentioned manifestations.

## 1. Introduction 

Systemic lupus erythematosus (SLE) is a systemic autoimmune disorder characterized by the production of various autoantibodies that cause damage to multiple organs involving the skin, joints, heart, lungs, kidneys, and central nervous system (CNS) [1]. However, the precise etiology remains unclear. SLE is characterized by hyper-reactivity of B lymphocytes, hyper-gammaglobulinemia, circulating immune complexes, and production of organ-specific and non-organ-specific autoantibodies. Moreover, dysregulated cellular immune responses are at times featured as lymphopenia and monocytosis. Numerous studies have shown that both T-cell activation and proinflammatory cytokine production are critically involved in SLE pathogenesis. 

Glucocorticoid-induced tumor necrosis factor receptor family-related protein (GITR) is a type I transmembrane protein belonging to the TNFR superfamily, and its cytoplasmic domain shares strong homology with a subgroup of the TNFR superfamily lacking the death domain, including CD27, CD134 (OX40), and CD137 (4-1BB). GITR is expressed predominantly on CD4^+^CD25^+^ regulatory T cells at high levels [2–4]. Moreover, other cells with regulatory activity, such as CD4^+^CD25^−^, CD8^+^CD25^+^, and CD8^+^CD28^−^ cells, express GITR at high levels [5]. However, its expression has also been detected on many cell types of both innate and adaptive immunity including monocytes, macrophages, neutrophils, dendritic cells (DCs), B cells, NK cells, and mast cells, and its expression level is increased after activation and during inflammatory or autoimmune processes [6–9]. GITR is activated by its ligand GITRL (TNFSF18), a type II transmembrane protein belonging to the TNF superfamily. GITRL is expressed on a subpopulation of T cells and monocytes [10, 11]. Notably, antigen-presenting cells and endothelial cells are found to express high levels of GITRL [12, 13]. The GITR/GITRL pathway has been shown to modulate DC function and promote T-cell-mediated immunity [14]. Recent studies have also indicated that the functional interaction of GITR with its cognate ligand GITRL delivers a potent costimulatory signal to enhance T-cell activation and cytokine production with significant implications for cancer immunotherapy [15–17]. Moreover, GITRL has been shown to modulate cytokine release and NK cell reactivity in chronic lymphocytic leukemia [18].

As a costimulatory molecule for CD4^+^ effector T-cell activation, GITR has been implicated in the development of autoimmune disease as revealed by recent studies on the murine model of collagen-induced arthritis (CIA) [19, 20]. Wang et al. showed that treatment of CIA mice with GITRL resulted in an earlier onset of arthritis with markedly increased severity of arthritic symptoms and joint damage, accompanied by significantly increased Th17 cells [21]. Furthermore, it was found that GITRL protein levels in the serum samples of rheumatoid arthritis (RA) patients were significantly higher than those in samples from healthy control subjects [21]. Notably, the increased levels of GITRL in RA patients were positively correlated with the DAS-28 scores of these patients [21]. However, it is currently unclear whether dysregulated GITRL expression is also involved in the development of other autoimmune diseases.

In this study, we sought to determine the possible involvement of GITRL expression in the development of SLE by examining the correlation of serum GITRL levels with disease activity and clinical manifestations in SLE patients.

## 2. Materials and Methods

### 2.1. Patients and Serum Samples

 The study group comprised 58 patients (54 women and 4 men) with a mean age of 30.6 ± 11.5 years. All patients were recruited from the Department of Rheumatology, The First Affiliated Hospital of Nanjing Medical University, China, between December 2011 and June 2012 and fulfilled the revised American College of Rheumatology criteria for SLE [22], and individuals with other rheumatic diseases, infections, or malignant tumors were excluded from the study. Sera were also collected from 30 healthy controls at the same hospital, and all recruited healthy controls were excluded from having any autoimmune diseases. There were no significant differences in the ages or sex ratios between the two groups. Clinical and laboratory information obtained at the time of serum sampling included age, gender, antinuclear antibodies (ANA), the titers of antidouble stranded (ds) DNA antibody, erythrocyte sedimentation rate (ESR), C-reactive protein (CRP), and the levels of IgG, IgM, and IgA, complement 3 (C3) and C4 and 24-hour urine protein. The clinical data from the patients were recorded in Table 1. Antinuclear antibodies (ANA) were detected by indirect immune fluorescence, and the titers of antidsDNA were detected by enzyme-linked immunosorbent assay (ELISA). Disease activity was assessed using the SLE Disease Activity Index (SLEDAI) and ranged from 0 to 34 (mean ± SD = 22.5 ± 12.02). The study was approved by the Research Ethics Committee of Jiangsu Province Hospital with informed consent from both patients and control group subjects.

 A volume of 5 mL peripheral venous blood was collected from each patient and control subject and allowed to clot at room temperature for 2 hours. Samples were then centrifuged for 10 min at 800 g. The serum samples were separated and frozen at −80°C until further analysis.

### 2.2. Determination of Serum GITRL Levels

Serum GITRL levels were measured using an ELISA kit (RayBiotech Inc.) according to the manufacturer's instructions. Briefly, serum samples (1 : 200 dilution) and standards were added to the wells. After incubation and washing, biotinylated antihuman GITRL antibodies were added, followed by incubation with HRP-conjugated streptavidin and color development with TMB substrate solution. The intensity of the color reaction was measured using a microplate reader (Bio-Rad, Beijing, China) at a wavelength of 450 nm. Concentrations of GITRL were determined by a standard curve according to the manufacturer's instructions.

### 2.3. Statistical Analysis

Data were presented as mean ± standard deviation unless specified otherwise. Statistical analysis was performed using SPSS for Windows (version 11.5). The Mann-Whitney rank sum test or Kruskal-Wallis tests were used to compare GITRL levels. The correlation between GITRL levels and various values were analyzed by Spearman's rank correlation coefficient. A value of *P* < 0.05 was considered statistically significant.

## 3. Results

### 3.1. Serum GITRL Levels Were Significantly Higher in Patients with SLE Than Normal Controls

In this study, 58 SLE patients and 30 healthy controls were recruited. There were no significant differences in both mean age and sex distribution between SLE patients and normal controls (Table 1). 

**Figure 1:**
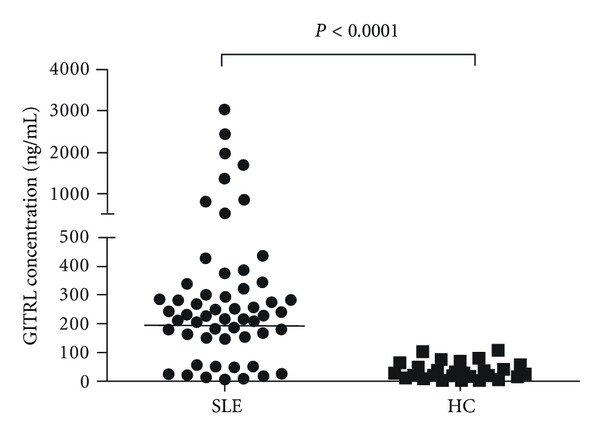
Comparison of serum GITRL levels between SLE and HC. Serum GITRL levels were significantly elevated in SLE patients versus HC. Each symbol represents an individual patient and healthy donor. Horizontal lines indicate median values. HC: healthy control; SLE: systemic lupus erythematosus.

To determine whether GITRL expression was dysregulated in SLE patients, we examined serum GITRL levels by ELISA. As shown in Figure 1, serum levels of GITRL were significantly higher in SLE patients than in healthy subjects (401.3 ± 79.96 ng/mL versus 36.59 ± 8.50 ng/mL; *P* < 0.0001), indicating that GITRL overexpression is probably involved in the pathogenesis of SLE. 

### 3.2. Serum GITRL Levels Were Markedly Higher in SLE Patients with Active Disease

**Figure 2:**
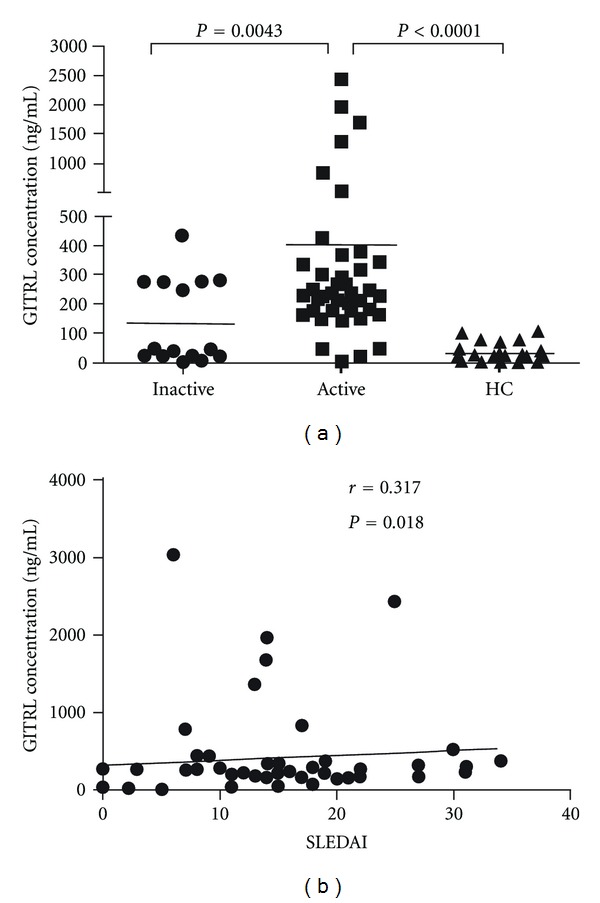
Comparison of serum GITRL levels among SLE patients with active disease and inactive disease as well as HC. (a) Serum GITRL levels were significantly elevated in SLE patients with active disease compared with those with inactive disease together with HC. (b) Serum GITRL levels were positively correlated with SLEDAI. Each symbol represents an individual patient and healthy donor; horizontal lines indicate median values. HC: healthy control; SLE: systemic lupus erythematosus.

We further grouped the SLE patients by active or inactive phase according to their SLEDAI scores. SLE patients were divided into the active group (SLEDAI score ≽8) and the inactive group (SLEDAI score <8; *n* = 15) [23]. As shown in Figure 2, serum GITRL levels in SLE patients with active disease were significantly higher than those with inactive disease (403.3 ± 81.23 ng/mL versus 136.3 ± 34.41 ng/mL; *P* = 0.0043), furthermore serum GITRL levels were positively correlated with SLEDAI (*r* = 0.317; *P* = 0.018), indicating a close correlation of increased GITRL expression with SLE disease activity.

### 3.3. Correlation of Serum GITRL Levels with Laboratory Values

**Figure 3:**
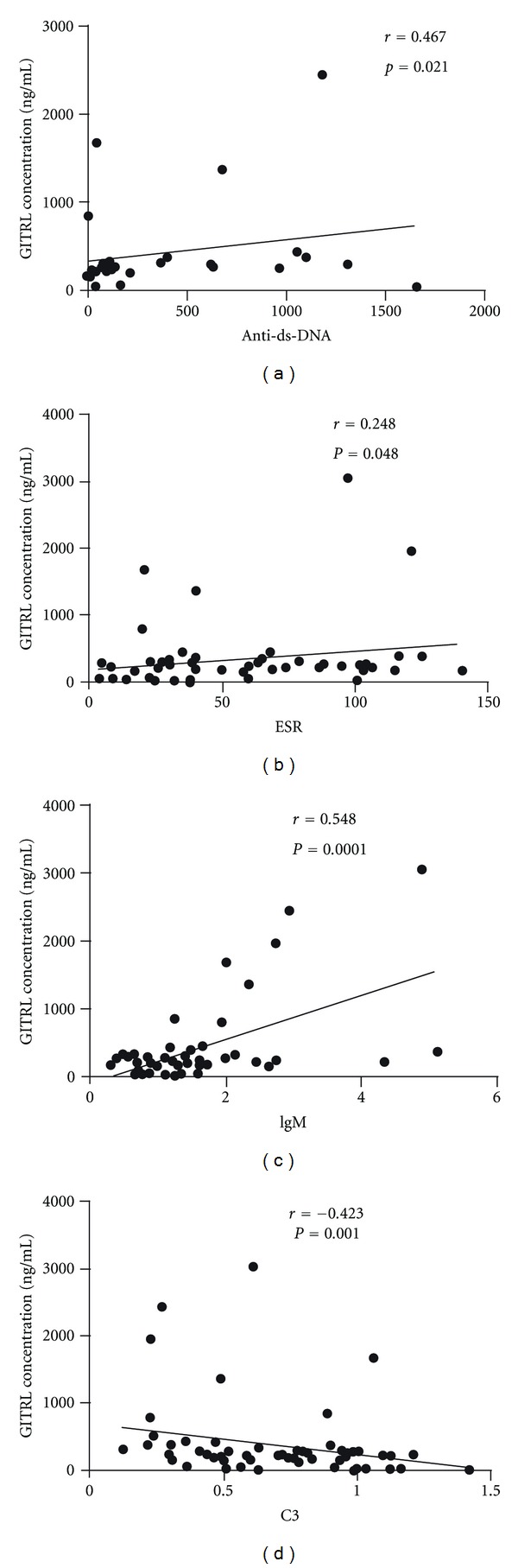
Correlation of serum GITRL levels with laboratory values. Each symbol represents an individual patient. (a) Positive correlation was observed between serum GITRL levels and the titers of antidsDNA antibody. (b) Positive correlation was also seen between serum GITRL levels and ESR. (c) Positive correlation was also observed between serum GITRL levels and IgM. (d) Negative relationship was observed between serum GITRL levels and C3. Anti-dsDNA antibody, antidouble stranded DNA antibody. C3: complement 3; ESR: erythrocyte sedimentation rate; IgM: immunoglobulin M.

To further determine the relationship between serum GITRL levels and laboratory test results including the titers of anti-dsDNA, ESR, CRP, C3, C4, and Ig levels, it was found that serum GITRL levels were positively correlated with the titers of anti-dsDNA antibody, ESR, and IgM (*r* = 0.467, *P* < 0.021; *r* = 0.284, *P* = 0.048; *r* = 0.548, *P* < 0.0001, resp.; Figure 3). Interestingly, there was a negative correlation between serum GITRL levels and C3 (*r* = −0.423; *P* = 0.001, Figure 3). However, no significant correlations were found between serum GITRL levels and CRP, C4, and ANA (Table 2).

### 3.4. Correlation of Serum GITRL Levels with Clinical Features in SLE

Serum GITRL levels were further compared among SLE patients with and without certain clinical features to assess the associations between serum GITRL levels and clinical manifestations. We did not detect any significant differences in serum GITRL levels between patients in the presence of fever, alopecia, arthritis, chest affection, anemia, thrombocytopenia, and leucopenia and those lacking the above-mentioned clinical manifestations (Table 3).

**Figure 4:**
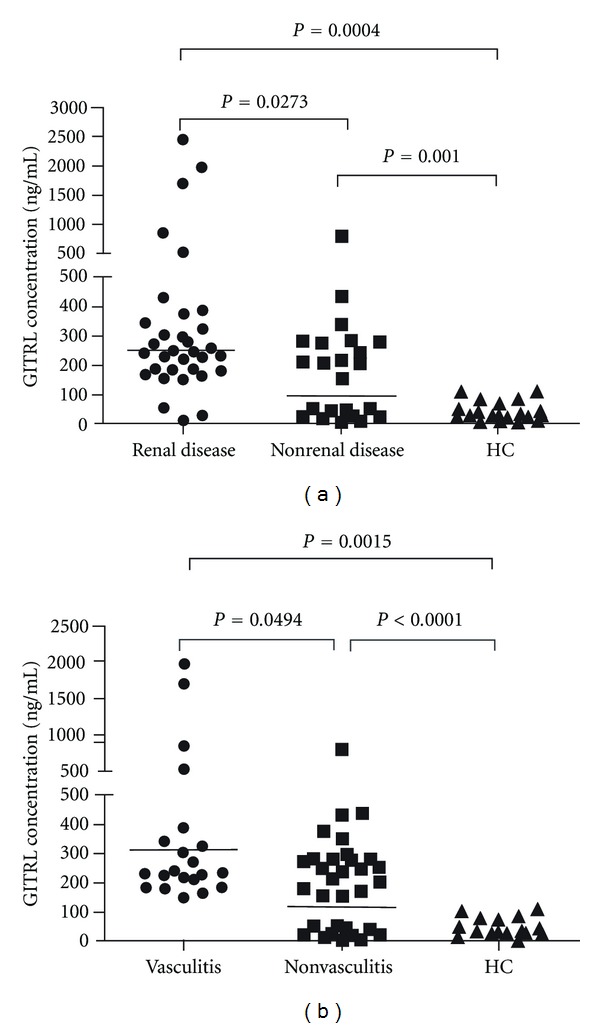
Elevated serum GITRL levels in SLE patients with organ damage. (a) Serum GITRL levels exhibited elevation in SLE patients with renal involvement (*n* = 33) relative to patients without renal involvement (*n* = 25) as well as HC. (b) Serum GITRL levels were higher in SLE patients with vasculitis (*n* = 24) than patients in the absence of vasculitis (*n* = 35) as well as HC. Each symbol represents an individual patient; horizontal lines indicate median values. GITRL: glucocorticoid-induced TNFR-related protein ligand; HC: healthy control; SLE: systemic lupus erythematosus.

However, we found that serum GITRL levels were significantly higher in patients with lupus nephritis and vasculitis compared with those patients without these manifestations as well as with normal controls (*P* = 0.0273; *P* = 0.0493, resp.; Figure 4), indicating a correlation of increased serum GITRL with renal damage and vasculitis in SLE patients.

## 4. Discussion

The pathogenesis of SLE involves complex interactions among genetic and environmental factors as well as the immune systems. SLE represents the classical prototype of systemic autoimmune disease in which loss of immune tolerance to self-antigens leads to activation and expansion of autoreactive lymphocytes, uncontrolled production of several autoantibodies, and release of inflammatory mediators that ultimately damage multiple organs. Numerous cytokines and costimulatory molecules are implicated in immune dysregulations, leading to autoimmune pathogenesis. In this study, we have revealed a close correlation of circulating GITRL levels with the disease activity in SLE patients. Our data have clearly shown that serum levels of GITRL are positively correlated with SLEDAI, the titers of anti-dsDNA antibody, renal involvement, and vasculitis in SLE. Here, we provide new evidence indicating the possible involvement of GITRL overexpression in the disease progression of SLE.

Many studies have showed the insufficiency or dysfunction of Tregs closely correlates with the development of autoimmune and inflammatory diseases as well as the interruption of immune homeostasis [24–26]. Pedroza-Gonzalez et al. showed that tumor Tregs upregulated the expression of glucocorticoid-induced GITR compared to Tregs in tumor-free liver tissue and blood. Meanwhile, treatment with soluble GITRL induced a decrease in the suppression mediated by the activated tumor-infiltrating Tregs and restores the proliferative capacity and cytokine production of CD4^+^CD25^−^ T cells [27]. A previous study by Crispin et al. reported a significant reduction of CD4^+^CD25^+^ Tregs in active SLE patients when compared to healthy controls and inactive patients [28]. Recent studies have demonstrated that the activation of GITR/GITRL pathway could stimulate the proliferation of effector T-lymphocytes and partially reverse the immunosuppressive function of CD4^+^CD25^+^ Tregs [29]. These results suggest an important role of GITR/GITRL activation in the loss of immune tolerance, especially in the dysfunction of Tregs. Future studies are needed to investigate whether and how GITR-GITRL pathway modulates the homeostatic regulation of Treg/Th17 cells during the pathogenesis of autoimmune diseases such as SLE. Our results from ELISA analysis have found higher levels of serum GITRL in the patients with active disease, consistent with previous findings that significantly elevated levels of GITRL are detected in RA patients with active disease [21]. Together, these results indicate a proinflammatory role of GITR/GITRL pathway in driving autoimmune progression. Although the source of the levels of serotype GITRL is currently unclear in this work, we speculate that activated dendritic cells could be one of the major cell types for GITRL overproduction in SLE patients, as indicated by recent findings that significantly increased expression of GITRL was detected in CD11c^+^ DCs during the development of experimental arthritis in mice [21]. Future studies are needed to examine whether DCs from SLE patients express increased levels of GITRL and to identify the signals that trigger the shedding of membrane GITRL from these cells. Furthermore, studies are required to verify whether treatment with glucocorticoids or immunosuppressive agents might exert their therapeutic effect on SLE patients by inhibiting the overexpression of GITR and GITRL proteins. 

In the current study, SLE patients are divided into active and inactive groups according to their SLEDAI scores [23]. We have found that serum GITRL levels in patients with active disease are significantly higher than those in patients with inactive disease as well as normal controls. It has been reported that treatment with GITR specific antibody or removal of T cells with high GITR expression can induce organ-specific autoimmune diseases in normal mice [30]. Recent evidence indicates that the costimulation of T cells through GITR signaling pathway may function *via* the induction of MAPK and NF-*κ*B activation [13]. Since SLE is an autoimmune disease characterized by massive abnormal immune response, it is generally assumed that dysregulation of immune T-cell tolerance occurs in both human and murine SLE. It is conceivable that elevated serum GITRL levels in SLE patients may be one of the possible factors which lead to aberrant immune response. Thus, our current findings indicate the involvement of GITR-GITRL in the disease activity of SLE, a notion further supported by our results on the correlation between serum GITRL levels with several laboratory values, such as the titers of anti-dsDNA antibody and ESR, IgM in SLE patients. Our data also reveal a negative relationship between serum GITRL levels and C3. Since C3 is one of complement components, participating in elimination of immune complexes through combination with immunoglobulins to disturb interaction of crystallizable fragment in space, its reduction and the elevation of anti-dsDNA antibody titers indicate the disease activity of SLE. 

In patients with SLE, most common clinical manifestations include alopecia, mucosal ulcer, arthritis, malar rash, anemia, thrombocytopenia, leucopenia, nephritis, and vasculitis [31]. Although we have not observed any correlation of serum GITRL levels with rash, alopecia, fever, chest affection, arthritis, anemia, thrombocytopenia, and leucopenia, we find that serum GITRL levels are significantly higher in patients with renal involvement and vasculitis when compared to the patients without the above-mentioned disease manifestations. La Cava et al. demonstrated that the production of autoantibodies by B cells in SLE patients could be interrupted *via* induction of Tregs since Tregs could inhibit the production of dsDNA antibodies by B cells *via* cell contact inhibition induced by membrane bound TGF-*β* and GITR molecules [32]. Benjamin et al. have also reported that CD4^+^CD25^+^ effector memory T cells expressing CD134 and GITR are closely associated with disease activity and their participation in Wegener's granulomatosis [33]. Thus, these findings also suggest that activation of GITR by GITRL may be involved in the development of nephritis and vasculitis in SLE. 

## 5. Conclusions

Our current data on the correlation of elevated serum GITRL with disease activity in SLE suggest that GITR-GITRL may participate in the pathogenesis of SLE. These findings provide new insights in understanding the disease pathophysiology of SLE. Further studies are needed to validate GITRL as a new biomarker to assess the disease activity of SLE.

## Figures and Tables

**Table 1:** Characteristics of SLE patients and the control subjects.

	SLE (*n* = 58)	Control (*n* = 30)
Age (years)	30.6 ± 11.5	29.7 ± 6.8
Sex (female/male)	54/4	27/3
Disease duration (months)	24.9 ± 40.9	—
Alopecia *n* (%)	13 (22.4)	—
Rash *n* (%)	34 (58.6)	—
Arthritis *n* (%)	11 (19.0)	—
Fever *n* (%)	15 (25.9)	—
Pleuritis *n* (%)	9 (15.5)	—
Anemia *n* (%)	28 (48.3)	—
Leukopenia *n* (%)	23 (39.7)	—
Thrombocytopenia *n* (%)	5 (8.6)	—
Current renal disease *n* (%)	33 (56.9)	—
Vasculitis *n* (%)	24 (41.4)	—
Anti-ds-DNA *n* (%)	34 (58.6)	—
Low C3 *n* (%)	35 (60.3)	—
Low C4 *n* (%)	30 (51.7)	—
ESR *n* (%)	41 (70.7)	—
CRP *n* (%)	19 (32.7)	—
24-hour urine protein *n* (%)	28 (48.3)	—
SLEDAI	0 to 34 (22.5 ± 12.02)	—

Abbreviations—SLE: systemic lupus erythematosus; SLEDAI: systemic lupus erythematosus disease activity index; C3: complement 3; C4: complement 4; CRP: C-reactive protein; anti-ds-DNA: anti-double stranded DNA antibody; ESR: erythrocyte sedimentation rate; SLE: systemic lupus erythematosus; values are expressed as mean ± standard deviation.

**Table 2:** Correlation between serum GITRL levels laboratory values.

Parameter	Correlation coefficient	*P* value
Anti-ds-DNA	0.467	0.021
ESR	0.284	0.048
CRP	−0.164	0.271
C3	−0.423	0.001
C4	−0.256	0.059
IgG	0.161	0.24
IgA	−0.099	0.484
IgM	0.548	0.0001
ANA	0.224	0.093
24-hour urine protein	0.057	0.713

SLEDAI: SLE disease activity index; anti-ds-DNA: anti-double stranded DNA antibody; C3: complement 3; C4: complement 4; CRP: C-reactive protein; ESR: erythrocyte sedimentation rate; IgG: immunoglobin G; IgA: immunoglobin A; IgM: immunoglobin M; ANA: antinuclear antibody.

**Table 3:** Serum GITRL level in SLE patients with or without clinical characteristics.

Clinical characteristic	Present	Absent	*P* value
	(*n*) Mean ± SD	(*n*) Mean ± SD	
Alopecia	(13) 437.2 ± 136.6	(45) 371.7 ± 90.76	0.6933
Rash	(34) 481.8 ± 116.3	(24) 251.2 ± 77.81	0.1052
Arthritis	(11) 503.9 ± 201.3	(47) 355.7 ± 81.38	0.5061
Fever	(15) 506.8 ± 162.8	(43) 327.8 ± 81.28	0.3339
Chest affection	(20) 516.9 ± 183.9	(38) 317.7 ± 64.74	0.3176
Anemia	(28) 472.2 ± 138.5	(30) 306.3 ± 70.83	0.2926
Leukopenia	(23) 273.7 ± 22.82	(35) 404.4 ± 88.34	0.1576
Thrombocytopenia	(5) 361.3 ± 134.3	(53) 388.8 ± 82.81	0.8669
Renal disease	(33) 418 ± 95.09	(25) 183.3 ± 38.32	0.0273*
Vasculitis	(24) 431.4 ± 106.7	(34) 201.6 ± 29.92	0.0494*

*P < 0.05, means significant difference.
